# The Role of Stress and Stress Adaptations in Determining the Fate of the Bacterial Pathogen *Listeria monocytogenes* in the Food Chain

**DOI:** 10.3389/fmicb.2016.01865

**Published:** 2016-11-23

**Authors:** Kerrie NicAogáin, Conor P. O’Byrne

**Affiliations:** Bacterial Stress Response Group, Microbiology, School of Natural Sciences, College of Science, National University of IrelandGalway, Ireland

**Keywords:** *Listeria monocytogenes*, general stress response, σ^B^, visible light, PrfA, virulence, RTE food safety

## Abstract

The foodborne pathogen *Listeria monocytogenes* is a highly adaptable organism that can persist in a wide range of environmental and food-related niches. The consumption of contaminated ready-to-eat foods can cause infections, termed listeriosis, in vulnerable humans, particularly those with weakened immune systems. Although these infections are comparatively rare they are associated with high mortality rates and therefore this pathogen has a significant impact on food safety. *L. monocytogenes* can adapt to and survive a wide range of stress conditions including low pH, low water activity, and low temperature, which makes it problematic for food producers who rely on these stresses for preservation. Stress tolerance in *L. monocytogenes* can be explained partially by the presence of the general stress response (GSR), a transcriptional response under the control of the alternative sigma factor sigma B (σ^B^) that reconfigures gene transcription to provide homeostatic and protective functions to cope with the stress. Within the host σ^B^ also plays a key role in surviving the harsh conditions found in the gastrointestinal tract. As the infection progresses beyond the GI tract *L. monocytogenes* uses an intracellular infectious cycle to propagate, spread and remain protected from the host’s humoral immunity. Many of the virulence genes that facilitate this infectious cycle are under the control of a master transcriptional regulator called PrfA. In this review we consider the environmental reservoirs that enable *L. monocytogenes* to gain access to the food chain and discuss the stresses that the pathogen must overcome to survive and grow in these environments. The overlap that exists between stress tolerance and virulence is described. We review the principal measures that are used to control the pathogen and point to exciting new approaches that might provide improved means of control in the future.

## Introduction

*Listeria monocytogenes* is a robust bacterial pathogen that is widely found in the environment. Its ability to persist in a diverse range of niches is underpinned by a sophisticated ability to sense and respond to the physicochemical stresses it encounters (Gandhi and Chikindas, 2007; O’Byrne and Karatzas, 2008). The term “stress” in this context is intended to mean any environmental perturbation that reduces the growth rate (a mild stress) or negatively impacts cell survival (a more severe stress). In general stress imposes an energy cost on cells because they have to invest resources in protection (e.g., homeostasis, synthesis of new macromolecules, repair and replacement of damaged components) if they are to continue to survive and grow. The stress responses deployed when stress is encountered confer on *L. monocytogenes* the ability to persist in soil environments, water, mammalian and avian feces as well as in food and food processing environments. They also allow it to make a successful transition from food into the gastrointestinal tract of mammalian hosts, which is a prerequisite for establishing infections in immunocompromised individuals. The stress tolerance mechanisms at its disposal allow *L. monocytogenes* to withstand acidic conditions, environments with low water activity, desiccation, low temperatures and bile. Many of these stress tolerance mechanisms are under the control of an alternative sigma factor called sigma B (σ^B^) whose role is to associate with RNA polymerase directing it to SigB promoters, which in turn leads to the reprogramming of the transcriptional profile of cells enabling the expression of protective functions (van Schaik and Abee, 2005; Chaturongakul et al., 2008; O’Byrne and Karatzas, 2008). The genes under the control of σ^B^, collectively known as the General Stress Response (GSR) regulon, are now well defined and many contribute to specific stress protective functions. Once within the host, an additional set of genes that allow cell invasion and systemic spread are expressed and these are regulated by a master transcriptional regulator called PrfA (Scortti et al., 2007). The roles of most of the virulence genes under PrfA control have well defined roles in the intracellular life cycle of the pathogen and indeed their study has fuelled the development of new areas of cell biology (Cossart and Toledo-Arana, 2008).

Although food-borne infections caused by *L. monocytogenes* are comparatively rare they are associated with unusually high mortality rates; typically 20–30% of clinical cases result in mortality. Immunocompromised individuals are most at risk, especially those with reduced T-cell immunity including elderly or very young patients, pregnant women, and individuals infected with HIV or on immunosuppressive treatment regimens (Lecuit, 2007). The organism is readily killed by normal cooking regimes including food processing treatments that use high temperatures (e.g., pasteurization). Therefore the main at-risk foods are the so-called ready-to-eat (RTE) foods, foods eaten without prior heating that have physio-chemical properties that can sustain the growth of *L. monocytogenes* (Chan and Wiedmann, 2008). Some of these foods include raw fruit and vegetables, dairy produce made with unpasteurised milk, minimally processed seafood, cold meats and pates (Farber and Peterkin, 1991; Lecuit, 2007). Although most countries enforce strict regulations on the tolerance for this pathogen in RTE foods its prevalence in the environment means that it is very difficult, if not impossible, to eradicate it from the food chain. Within Europe, if a product is capable of supporting growth, the producer must be able to demonstrate that levels of *L. monocytogenes* will not increase higher than 100 CFU/g over the course of the shelf life by means of a challenge study. However, if a RTE product is not capable of supporting growth, levels must not exceed 100 CFU/g during shelf life (European Commision, 2005). This differs from regulation in the US, where absence of *L. monocytogenes* is required in all RTE products (FSIS, 2014).

In this review, we discuss the route that *L. monocytogenes* can use to enter the food supply chain and discuss the behavior of the pathogen in foods. We outline the key stresses that *L. monocytogenes* must overcome to survive and grow in RTE foods and discuss the main protective systems that this pathogen uses to defend itself. The involvement of σ^B^ and the GSR regulon in these responses is a particular focus of the review. Finally, we discuss traditional control measures used to reduce the risk of *L. monocytogenes* contamination of foods as well as some more innovative approaches that are currently being developed.

## Entry of *L. monocytogenes* into the Food Chain

### Soil

**FIGURE 1:**
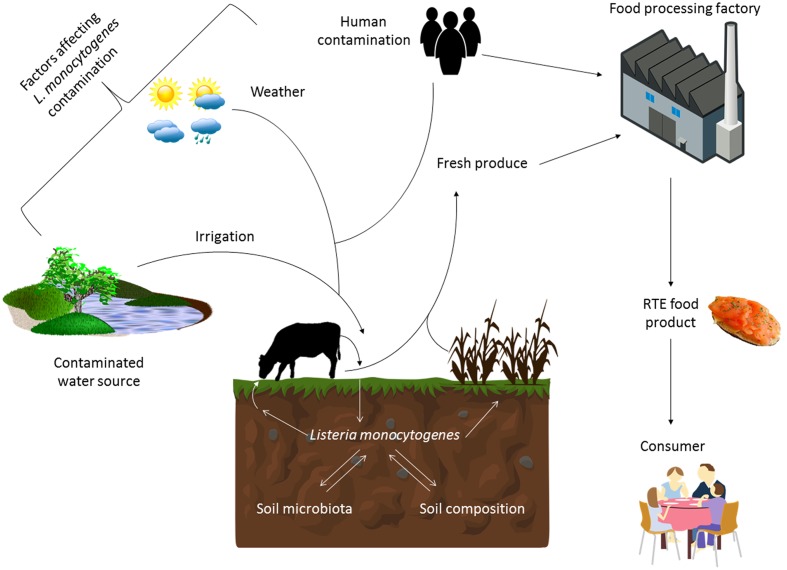
**Factors influencing the survival and transmission of *Listeria monocytogenes* in the environment and food chain.** The survival of *L. monocytogenes* in the soil is influenced by factors such as the composition of the soil and the competing microbiota present. Its presence in this environment is also influenced by weather events (sunshine and rainfall), irrigation from contaminated sources, as well as human and animal fecal contamination. Therefore agricultural produce can be contaminated with this pathogen at the point of harvest. This can introduce the pathogen into the food processing environment or the produce can become contaminated there if adequate cleaning and decontamination practices are not in place. Ready-to-eat food produce that can support the growth of *L. monocytogenes* is a particular risk to the consumer, especially those that are immunocompromised.

During the 1970’s it was suggested that soil was a natural environment for *L. monocytogenes* (Welshimer and Donker-Voet, 1971; Weis and Seeliger, 1975). However, more recent studies have suggested that soil contamination by the organism may come from other sources such as sewage, animal manure and decaying plant vegetation (Fenlon et al., 1996). Many studies have investigated the survival of *L. monocytogenes* in soil and have observed that the foodborne pathogen can survive over a period of time, although, soil type, water content, pH, and temperature can all have an influence on the rate of survival (**Figure 1**; Ivanek et al., 2009; McLaughlin et al., 2011). For example, Locatelli et al. (2013) found that survival of *L. monocytogenes* was higher in fine soil with high clay content, which they suggest has a higher number of pores for protection against predation by protists and also has a cation content that is more compatible with long term survival.

Microflora within the soil can highly affect the survival of *L. monocytogenes* (**Figure 1**). Interactions between *L. monocytogenes* and different types of protozoa have previously been demonstrated (Ly and Muller, 1990; Zhou et al., 2007; Pushkareva and Ermolaeva, 2010). Sterilization of soil can lead to an increase in growth of *L. monocytogenes* suggesting that the microflora of the soil such as bacteriophage or protozoa have an effect on persistence of the bacterium, although this effect has not yet been fully explained. McLaughlin et al. (2011) confirmed that the microbiota of the soil plays an important role on survival. In their study, they partially reconstituted sterile soil with culturable aerobic components of the soil microbiota and observed that this lead to a decrease in survival at later time points in the experiment. They discuss the possibility that this decrease may be due to competition by different microflora for nutrients within the soil. Other factors which may affect the survival of the organism in soil include chemical properties as well as geographical and meteorological influences (Ivanek et al., 2009; Strawn et al., 2013). For example, Weller et al. (2015b) examined temporal factors (irrigation and rainfall) leading to contamination of pre-harvest spinach (**Figure 1**). There was a greater chance of isolating *L. monocytogenes* after irrigation than rainfall and this chance was highest within 24 h of the event (Weller et al., 2015b). Other studies have confirmed similarly that irrigation is a risk factor for contamination of pre-harvest foods (Genereux et al., 2015; Weller et al., 2015a). This is often due to the contamination of the water source used for irrigation of the fields (Strawn et al., 2013; Genereux et al., 2015). Along with irrigation, the use of manure as a fertilizer can increase isolation of *L. monocytogenes* from produce production sites (Watkins and Sleath, 1981; Fenlon et al., 1996; Garrec et al., 2003). This is not surprising as animals are known reservoirs of the bacterium (Fenlon et al., 1996; Esteban et al., 2009; Mohammed et al., 2010).

Spatial factors such as proximity to urban areas, farms or water sources can lead to higher detection of *L. monocytogenes* (Sauders et al., 2012; Strawn et al., 2013; Weller et al., 2015b). One study conducted in New York State found that incidence of *L. monocytogenes* was much higher in samples taken from farms compared to a natural environment (an undeveloped area with minimal human presence) suggesting that the presence of humans and animals is highly associated with isolation of *L. monocytogenes* (Chapin et al., 2014).

### Seawater

Many studies have shown that water sources such as rivers, ponds and creeks can act as reservoirs for *L. monocytogenes* (Schaffter and Parriaux, 2002; Lyautey et al., 2007; Linke et al., 2014). However, one environment which has been considered to a much lesser extent is seawater. As isolation of this foodborne pathogen has been associated with seafood (Colburn et al., 1990; Johansson et al., 1999; Gonzalez et al., 2013; Leong et al., 2015), it may be a source of contamination worth considering. As with rivers, it is possible that effluent and land run off may increase levels of contamination by this microorganism in coastal waters (Watkins and Sleath, 1981; Fenlon et al., 1996). Some studies have shown isolation of *L. monocytogenes* from marine environments (Colburn et al., 1990; Motes, 1991; Rorvik et al., 2000; Rodas-Suarez et al., 2006). Motes (1991) found that *Listeria* spp. including *L. monocytogenes* could be isolated from fresh seafood and their harvest waters, suggesting that *L. monocytogenes* can survive in seawater for a period of time. Colburn et al. (1990) also isolated *L. monocytogenes* from samples taken from an estuary and a bay in California. However, while some studies don’t dispute that *L. monocytogenes* can be isolated from water, they do disregard it as an important source of contamination within fish farms (Jemmi and Keusch, 1994; Rorvik et al., 2000). More recent studies have also shown the survival of the pathogen in seawater, although many of these report that the survival of *L. monocytogenes* is strain and temperature dependent with lower temperatures correlating with higher survival (Bremer et al., 1998; Hsu et al., 2005; Hansen et al., 2006). However, besides temperature, other factors must also be considered for the survival of *L. monocytogenes* in seawater. Those factors include osmotic stress, predation by protozoa, nutrient availability, and solar irradiation (Smith et al., 1994; Tedetti and Sempere, 2006).

### Food Processing Environments

Within food production facilities, it is known that *L. monocytogenes* can survive over long periods of time; however, the source of contamination is often unknown (**Figure 1**). Persistence is often defined as a particular subtype re-isolated from the same environment over an extended period of time (Carpentier and Cerf, 2011; Ferreira et al., 2014). However, it is often difficult to determine whether a particular strain is persisting within an environment such as a food processing environment or if the strain is being reintroduced into the facility at different times. It is also disputed as to whether a genotype associated with persistence exists or whether *L. monocytogenes* can colonize specific favorable niches within a processing environment and therefore “persist” over a longer period of time (Carpentier and Cerf, 2011; Ferreira et al., 2014). Studies have compared phenotypic characteristics that cause strains to persist compared to non-persistent strains (Lunden et al., 2008; Ringus et al., 2012; Magalhaes et al., 2016). One inherent limitation of this sampling process is that only a subset of the population is sampled, and that persistent clones may be missed on multiple sampling occasions. Therefore, categorizing strains as non-persistent can be difficult as it may happen that a persistent strain was only isolated sporadically in a study (Ferreira et al., 2014). Another challenge is that apparent persistence could be caused by the repeated introduction of the same strain to a food production facility, which could happen if contaminated personnel, equipment or product serve as a vector to continually introduce the same strain from some reservoir outside the plant.

Different studies have been conducted to investigate the main sources of contamination within food processing facilities (Johansson et al., 1999; Hansen et al., 2006; Leite et al., 2006; Ho et al., 2007; Chen et al., 2010a; Rivoal et al., 2010). Hansen et al. (2006) found that there was evidence of strains isolated from the outside environment also being identified within fish slaughterhouses. Other studies have shown that operators within a facility or different pieces of equipment may also be considered sources of contamination (Leite et al., 2006; Lomonaco et al., 2009; Chen et al., 2010a). Lomonaco et al. (2009) isolated *L. monocytogenes* from locker rooms, hallways and toilets within a gorgonzola producing facility suggesting the possibility that personnel within the factory contributed to the problem of contamination. Chen et al. (2010b) found that water used to chill fish products along with a weighing table were important sources of contamination within their facility.

It is still disputed as to whether seasonal variation has a contributing role in the isolation of *L. monocytogenes* from food processing environments. Many studies show no correlation between seasonal variation and occurrence of *L. monocytogenes* (Garrec et al., 2003; Ho et al., 2007; Esteban et al., 2009; Mohammed et al., 2010; Leong et al., 2014) but others have disputed these findings by showing a link between the two (Rivoal et al., 2010).

## Stresses Encountered In Food

### Osmotic Shock

As salt is widely used in the preservation of food, osmotic stress is an important stress that *L. monocytogenes* must overcome to survive within many foods. This foodborne pathogen can survive salt concentrations as high as 3 M NaCl (Cole et al., 1990). It has been suggested that *L. monocytogenes* has a so-called primary and secondary response to osmotic shock. The primary response involves the influx of K^+^ and glutamate into the cell, while the secondary response involves the uptake of small molecules known as compatible solutes (Kallipolitis and Ingmer, 2001; Brøndsted et al., 2003). These methods of combating osmotic shock play a role in helping the bacterium to restore turgor pressure, cell volume and also help to stabilize cell protein structure and function (O’Byrne and Fraser, 2000; Sleator et al., 2003).

*Listeria monocytogenes* accumulates the compatible solutes, glycine betaine, and carnitine, in hyperosmotic environments (Fraser et al., 2000; Sleator and Hill, 2001; Wood et al., 2001; Sleator et al., 2003). These solutes can often be found in different foods, with glycine betaine commonly found in foods of plant origin and carnitine from foods of animal origin (Sleator et al., 2003). The presence of these osmolytes in foods can help to enhance the growth of *L. monocytogenes* in the presence of hyperosmotic conditions. Besides these main osmolytes, other compatible solutes including proline, proline betaine, acetylcarnitine, gamma-butyrobetaine and 3-dimethylsulfonioporpionate have also been found to help the growth of *L. monocytogenes* in osmotic stress conditions (Bayles and Wilkinson, 2000). Uptake of compatible solutes occurs via three main transporters, Gbu, BetL, and OpuC (Sleator et al., 1999; Fraser et al., 2000; Gerhardt et al., 2000; Angelidis et al., 2002; Angelidis and Smith, 2003b). BetL or Betaine Porter I is one of two systems involved in the transport of glycine betaine into the cell (Gerhardt et al., 1996; Sleator et al., 1999) and is dependent on the presence of Na^+^ (Gerhardt et al., 1996). Gbu, the second system involved in betaine uptake is an ATP dependent transporter which can be activated independently of Na^+^ in response to osmotic shock by excess sucrose or KCl (Ko and Smith, 1999; Gerhardt et al., 2000). Finally OpuCA has been characterized as a carnitine transporter (Fraser et al., 2000, 2003). Deletion of genes encoding these transporters leads to an increase in generation time of the bacteria in the presence of hyperosmotic stress when incubated with glycine betaine, and carnitine (Angelidis and Smith, 2003a). Interestingly, SigB promoter sites have been identified upstream of each of these genes and deletion of σ^B^ leads to reduced survival in response to high salt concentrations (Sleator et al., 1999; Fraser et al., 2003; Cetin et al., 2004). Further studies have shown that *opuC* and *gbu*A are under the control of σ^B^, but despite the presence of the putative SigB dependent promoter site upstream of *betL*, this gene does not appear to be under SigB control. Utratna et al. (2011) showed that transcription of *opuC* in response to osmotic shock occurred in a transient manner and the level of σ^B^ activity observed also appeared to be proportional to the level of osmotic stress encountered.

Along with overcoming osmotic upshock, some bacteria have mechanisms to deal with hypoosmotic conditions. Mechanosensitive channels can allow the controlled release of osmolytes and water from the cell to aid the survival of a rapid increase in turgor pressure that occurs during osmotic downshock (Wood et al., 2001). Not much information is known about the existence of these channels in *L. monocytogenes* but two genes, *lmo1013* and *lmo2064*, have been identified as having homology to genes encoding mechanosensitive channels in *Escherichia coli* and *Streptococcus pneumoniae* (Sleator et al., 2003). Rapid efflux of osmolytes, glycine betaine, and carnitine, has also been observed in *L. monocytogenes* cultures exposed to hypoosmotic conditions providing evidence for the presence of systems involved in downshock survival (Verheul et al., 1997).

*Listeria monocytogenes* also exhibits an adaptive response to NaCl known as osmoadaptation, where treatment of cells with a sub-lethal level of NaCl can offer increased survival following further exposure to lethal salt concentrations (Faleiro et al., 2003). A cross protection between osmotolerance and other stresses has also been confirmed. Schmid et al. (2009) found that *csp* genes are upregulated in the presence of either cold shock or osmotic shock. Deletion of some of these genes can lead to stunted growth when treated with low temperatures or high salt concentrations leading this group to hypothesize that the use of the CSP proteins may help to offer cross protection between osmotolerance and cold shock or vice versa depending on the condition encountered first by the bacterium (Schmid et al., 2009).

### Cold Shock

*Listeria monocytogenes* is capable of growth at temperatures as low as -0.4°C (Walker et al., 1990). Various studies have demonstrated growth of this foodborne pathogen in different foods at refrigeration temperatures. However, at these temperatures the doubling time of the bacterium can be up to 50 h or more (Angelidis and Smith, 2003a). During an encounter with cold temperatures, bacterial membranes become more rigid, the rate of enzymatic reactions reduces and the level of uptake and transport of molecules is also decreased (Graumann and Maraheil, 1996). The bacterium must modulate its gene expression to mitigate the effect of these physical changes. Changes in expression usually occur for genes involved in cell membrane function, lipid, carbohydrate and amino acid synthesis, ribosomal structure and biogenesis and motility (Chan et al., 2007b; Cordero et al., 2016).

During exposure to cold temperature, one of the methods used by *L. monocytogenes* to combat cold shock is the accumulation of low molecular weight solutes such as glycine betaine, and carnitine. High amounts of these solutes are found in various foods (Zeisel et al., 2003; Demarquoy et al., 2004), which may help to promote the survival and growth of this pathogen in foods at refrigeration temperatures. The generation time of *L. monocytogenes* reduces by more than 20 h at 4°C when cells are incubated in the presence of compatible solutes (Angelidis and Smith, 2003a). The BetL glycine betaine transporter (see Osmotic Shock) does not seem to be involved in cryotolerance (Sleator et al., 2003). Chan et al. (2007b) identified increased expression of both Gbu and OpuC but not BetL in response to cold shock, while a metabolomics study also showed increased quantities of glycine betaine, and carnitine present within *L. monocytogenes* when grown at 8°C compared to 37°C (Chan et al., 2007b; Singh et al., 2011). The increase in solute levels within the cell may help to decrease loss of intracellular water from the cell when temperatures drop.

A number of studies have investigated the role of σ^B^ in adaptation to cold stress, but the data show conflicting results. It seems likely that survival, during exposure to cold temperatures, is controlled in a manner that is at least partly σ^B^-dependent. For example, Chan et al. (2007a) demonstrated that while some cold-induced genes were under σ^B^ control (*opuCA)* or were preceded by a σ^B^ dependent promoter site, they could be activated in a σ^B^ independent manner at 4°C indicating that genes responding to cold shock may be partially under σ^B^ control. They also showed that a mutant lacking *sigB* did not have reduced growth at 4°C compared to the wildtype (Chan et al., 2007a). Utratna et al. (2014) showed σ^B^ does not play a large role in survival at low temperatures. They also showed that σ^B^ could be activated at 4°C in a manner that was independent of RsbV without levels of RsbW being affected (Utratna et al., 2014). Other systems that have been suggested to play a role in adaptation to cold stress include the two component regulatory systems, YycGF and LisRK (Pontinen et al., 2015). Transcript levels of the *yycF* gene were shown to be increased at 4°C (Chan et al., 2007b) and Pontinen et al. (2015) suggested that YycF was more involved in survival of initial cold stress than acclimation over time, whilst LisRK seems to be more involved in acclimation.

### Low pH

*Listeria monocytogenes* can often encounter acidic conditions either in food matrices or within the gut of the host. These acidic conditions can arise from either weak organic acids such as lactate, benzoate, acetate or sorbate, or by strong acids like hydrochloric acid. Once *L. monocytogenes* enters the host following the ingestion of contaminated food, it encounters acidic conditions firstly within the stomach but also within the vacuole of the macrophage phagosome after intracellular uptake. The bacterium possesses a variety of different mechanisms including the adaptive acid tolerance response (ATR), the Glutamate Decarboxylase (GAD) system and the Arginine Deaminase (ADI) system to help it overcome these acidic environments (Davis et al., 1996; Cotter et al., 2001a; Ryan et al., 2009).

#### Acid Tolerance Response (ATR)

Davis et al. (1996) first confirmed the presence of the Adaptive ATR in *L. monocytogenes.* This study showed that when exponential cells were pre-exposed to a sub-lethal pH (pH 5.0) for 1 h prior to exposure to a lethal pH (pH 3.0), cells exhibited a much higher survival rate compared to unexposed cells (Davis et al., 1996). The ATR results from pre-exposure to cells at a sub-lethal pH, typically between pH 5-6, before exposure to more lethal acids (Davis et al., 1996; Skandamis et al., 2012). Some studies have shown how this response can help *L. monocytogenes* survival on low pH foods (Gahan et al., 1996; Skandamis et al., 2012) while other studies also showed that this protective effect could be extended to other stresses such as heat and osmotic shock (O’Driscoll et al., 1996; Skandamis et al., 2009). Ferreira et al. (2003) have investigated whether the GSR has a role in the ATR. They suggest that while an isogenic Δ*sigB* mutant survives less than the parent strain after being pre-exposed to sub-lethal pH, survival increases after pre-exposure suggesting that there are other σ^B^-independent mechanisms working on survival against acid (Ferreira et al., 2003).

#### Arginine Deaminase (ADI) System

The ADI system is involved in enhanced survival at low pH in a variety of Gram-positive microorganisms including *L. monocytogenes* (Cunin et al., 1986). The system works by converting molecules of arginine into ornithine using three enzymes encoded for by the *arcABC* operon. A membrane antiporter ArcD, transports a molecule of arginine into the cell which is then converted to ornithine, CO_2_, ammonia and ATP. Ornithine is then transported back out of the cell in exchange for another molecule of arginine. During this process, the by-product ammonia can associate with intracellular protons to form NH_4_^+^ and this leads to an increase of the cytoplasmic pH (Cunin et al., 1986). Many studies have investigated the role of the ADI system in acid survival in *L. monocytogenes* (Ryan et al., 2009; Chen et al., 2011; Cheng et al., 2013). Ryan et al. (2009) first showed the presence of a functional ADI system within *L. monocytogenes* and demonstrated that it is implicated in survival at low pH and virulence *in vivo*. They also identified ArgR as a regulator of the ADI system. Another study showing the role of Lmo0036 (ArcB) in acid tolerance also confirms the role of the ADI system (Chen et al., 2011). Interestingly, the transcription of *arcA* and *argR*, have been shown to be both SigB and PrfA-dependent (Ryan et al., 2009). Hain et al. (2008) and Bowman et al. (2010) also identified *arcA* as being under SigB control, whilst Milohanic et al. (2003) identified it as being controlled by PrfA suggesting a role for the ADI system in both stress response and virulence.

#### Glutamate Decarboxylase (GAD) System

Another system identified in *L. monocytogenes* to help to maintain pH homeostasis within the cell is the GAD system, known to be important for survival both within synthetic gastric fluid, infection in mouse models and also in acidic foods (Cotter et al., 2001a,b; Feehily et al., 2014). It works to increase the internal pH of the organism in the presence of extracellular acidic conditions. However, it has been suggested that the GAD system is only responsible for survival of strong acidic conditions (below pH 4.5) and does not have a role to play in tolerance to weak acids (Heavin et al., 2009; Karatzas et al., 2010). The process works by the utilization of glutamate, which is present in all foods and living organisms. Under acidic conditions an extracellular molecule of glutamate is taken up by an antiporter (GadT) and then converted into γ-aminobutyric acid (GABA) by a decarboxylase enzyme, GadD. GABA is then exported back out of the cell in exchange for another molecule of glutamate. The decarboxylation process consumes one proton, thereby leading to an increase in intracellular pH (Lund et al., 2014). In *L. monocytogenes*, there are five genes involved in the Gad system, three genes encoding glutamate decarboxylases (GadD1, GadD2, and GadD3) and two encoding antiporters (GadT1 and GadT2). These genes are arranged into three operons, *gadD1T1*, *gadD2T2*, and g*adD3* (Cotter et al., 2005). GadD1T1 seems to be required for growth at mild pH conditions while GadD2T2 is important in more severe acidic conditions. GadD2T2 and GadD3 have been shown to be at least partially under σ^B^ regulation (Kazmierczak et al., 2003; Wemekamp-Kamphuis et al., 2004), but little more is known about the regulation of the GAD system in *L. monocytogenes*.

### Nisin

Different antimicrobial compounds including bacteriocins have been studied extensively over the past decades as a method of controlling bacterial contamination within food products. Some examples of antimicrobials which have been proven to be active against *L. monocytogenes* include lauric arginate, chitosan, pediocin, and nisin (Kaur et al., 2013; Kang et al., 2015). Nisin is one of the most common antimicrobials used in the food industry especially within dairy products and acidic foods (Delves-Broughton et al., 1996). It is a bacteriocin that is produced by the lactic acid bacterium, *Lactococcus lactis*. Compared to other bacteriocins, nisin has been shown to be most effective at reducing numbers of *L. monocytogenes* (Kaur et al., 2013). However, when used in combination with other antimicrobials, levels of inhibition increase further (Tokarskyy and Marshall, 2008; Kaur et al., 2013). It has also been observed that *L. monocytogenes* isolates can develop resistance to nisin, which is potentially a worrying prospect for the food industry (Gravesen et al., 2002). Cross resistance can also develop between bacteriocins meaning that combinations of different bacteriocins may not always be feasible (Kaur et al., 2013).

The antimicrobial effect of nisin involves interference with cell wall biosynthesis, disruption of the cell membrane by the formation of pores and consequent disruption of cell membrane associated processes (Bruno et al., 1992; Abee et al., 1994). It has been suggested that resistance of *L. monocytogenes* to nisin may arise due to changes within the cell wall composition which stops the bacteriocin from gaining access to the cell and therefore increasing survival (Kaur et al., 2012). Different systems including two component regulatory systems and the GSR have been implicated in *L. monocytogenes* resistance to nisin. Kang et al. (2015) showed that a mutant deficient in the response regulator VirR had a greater loss of membrane integrity compared to the wild-type strain, while Begley et al. (2006) found that a *sigB* mutant had decreased growth and survival in response to nisin. However, Palmer et al. (2009) reported data which conflicted these results. They suggested that σ^B^ contributes to nisin resistance in *L. monocytogenes* but only when it is deleted in a background lacking another alternative sigma factor, SigL (σ^L^). When Δ*sigB* is solely deleted, growth and survival actually increases in response to nisin. These data suggest that both σ^B^ and σ^L^ have a role to play in nisin resistance in *L. monocytogenes* (Palmer et al., 2009). Thus the actual role of σ^B^ in the response of *L. monocytogenes* to nisin has yet to be determined.

### Light

**FIGURE 2:**
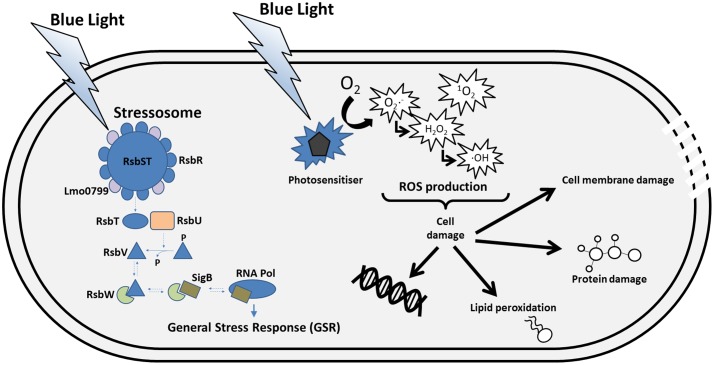
**Sensing and consequences of blue light in the cell.** *L. monocytogenes* senses blue light via the photoreceptor Lmo0799, which is thought to be part of the stressosome complex. Other stress signals can be “sensed” by RsbR and its paralogs in the stressosome. The light signal causes a conformation change in the stressosome complex that triggers the release of RsbT, which in turn activates a phosphatase called RsbU. RsbU acts on to dephosphorylate RsbV, an anti-anti sigma factor that antagonises the anti-sigma factor RsbW. The interaction between RsbV and RsbW liberates SigB to interact with RNA polymerase and consequently leads to the transcription of the general stress response (GSR) regulon. Blue light can also interact with photosensitisers in the cell which, in the presence of oxygen, can lead to the production of reactive oxygen species (ROS). The ROS produced can cause damage of cellular macromolecules, which in extreme cases results in cell death.

Within environments where *L. monocytogenes* can persist, the bacterium can encounter varying amounts of light. Light has previously been used as a method of bacterial decontamination both within clinical environments and on food products (Ozer and Demirci, 2006; Maclean et al., 2010; Hosein et al., 2016; Xu and Wu, 2016) and therefore may be useful as a means of controlling *L. monocytogenes* contamination within the food industry. Recently it has been shown that blue light triggers the activation of the GSR within *L. monocytogenes* and therefore should be considered as a stress for the bacterium (Ondrusch and Kreft, 2011; Tiensuu et al., 2013). It is known that many bacteria have light sensing mechanisms which help them to overcome this stress. Within *Bacillus subtilis*, a light sensing protein YtvA has been discovered (Losi et al., 2002; Avila-Perez et al., 2006, 2009). This protein is present in a stress sensing complex known as the stressosome which is composed of the proteins RsbR and its paralogs as well as RsbS and RsbT (**Figure 2**; Gaidenko et al., 1999; Kim et al., 2004b; Hecker et al., 2007; Marles-Wright et al., 2008; Jurk et al., 2013). The stress signals are thought to be sensed by the protruding N- termini of these sensory proteins and are transduced into the core of the stressosome (Marles-Wright et al., 2008). This leads to a signaling cascade downstream of the stressosome which ultimately leads to the activation of σ^B^ in response to the stress (**Figure 2**; Avila-Perez et al., 2009). While it has not yet been confirmed in *L. monocytogenes*, it is hypothesized that a similar stress-sensing complex exists. Within *L. monocytogenes*, the paralogs of RsbR include, Lmo0799, Lmo0161, Lmo1642, and Lmo1842 (Ondrusch and Kreft, 2011; Heavin and O’Byrne, 2012). Although it is not clear what stress signals most of these proteins sense, Lmo0799, a homolog of YtvA in *B. subtilis*, has been confirmed as a blue light photoreceptor (Ondrusch and Kreft, 2011). Mutants lacking Lmo0799 have been shown to have similar phenotypes to a Δ*sigB* mutant with higher levels of motility in the presence of blue light and have lost the ability to form rings in response to cycles of light and dark (Ondrusch and Kreft, 2011; Tiensuu et al., 2013). The protein consists of an LOV (Light, Oxygen, and Voltage) domain at its N-terminus and a STAS domain at its C-terminal region. LOV domain proteins belong to the Per-Arnt-Sim (PAS) superfamily and can bind a flavin cofactor such as FMN, to facilitate light sensing (Christie et al., 1999; Herrou and Crosson, 2011). During light exposure it is thought that a covalent bond forms between a thiol residue of a conserved cysteine residue at position 56 of the Lmo0799 protein and the FMN molecule found in the binding pocket of the protein (Chan et al., 2013). Recently, O’Donoghue et al. (2016) constructed a mutant with a missense mutation, changing Cys56 to an alanine. When tested in response to light this mutant showed similar phenotypes to both Δ*sigB* and Δ*lmo0799* suggesting that this residue is indeed required for light sensing by this protein (O’Donoghue et al., 2016). Interestingly, while it has been shown that σ^B^ is activated in response to light, it has also been demonstrated that virulence genes have also been activated. Ondrusch and Kreft (2011) investigated the transcription levels of the internalin genes (*inlA* and *inlB*) that are involved the in invasion of *L. monocytogenes* into epithelial cells. Transcription of both *inlA and inlB* was increased in response to blue light in combination with 0.3 M NaCl and invasion into Caco-2 enterocyte-like human cells was also increased under these conditions. These data suggest that along with activating the stress response, blue light may also play a role in activation of virulence genes within *L. monocytogenes.*

## Implications For Food Safety

As RTE foods are of major concern for contamination by *L. monocytogenes*, it is beneficial to investigate how the bacterium behaves in such foods. This section looks at the response of stress related and virulence genes within different food matrices and how these could prime the bacterium for survival within the host. We also discuss the relationship between σ^B^ and PrfA within the host and how this may aid survival and pathogenesis.

### Behavior of *L. monocytogenes* within Food Matrices

Studies have been conducted to investigate whether the transcriptional response of stress related and virulence genes of *L. monocytogenes* differ within various food matrices (Olesen et al., 2010; Bae et al., 2011; Alessandria et al., 2013). Within RTE foods, the bacterium encounters many of the stresses discussed in this review. Therefore, transcriptional studies can provide information on genes involved in allowing the survival of *L. monocytogenes* in the presence of these stresses *in situ*. Importantly, virulence of *L. monocytogenes* has been shown to be heterogeneous between strains and between food matrices (Duodu et al., 2010; Olesen et al., 2010; Rantsiou et al., 2012a,b; Hadjilouka et al., 2016). Virulence genes have been reported to be more highly induced under laboratory conditions than in food matrices, (Olesen et al., 2010; Rieu et al., 2010) and this was confirmed by a study which tested the effects on mice fed with broth cultures compared to contaminated food (Mahoney and Henriksson, 2003). The mice that were fed with fermented salami batter spiked with *L. monocytogenes* had a lower rate of infection than mice intragastrically challenged with a broth culture (Mahoney and Henriksson, 2003). However, mixed results have been found for the levels of *sigB* transcription when comparing broth cultures to food matrices. When grown on RTE deli turkey meat, transcriptional levels of *sigB* and related genes remained unchanged when compared to cultures grown in BHI broth (Bae et al., 2011). Rantsiou et al. (2012b) observed that *sigB* transcript levels are generally upregulated in food matrices incubated at low temperature compared to BHI broth at 37°C, while in contrast Olesen et al. (2010) showed that the level of *sigB* transcription is increased in BHI broth compared to standard liver pâté. Somewhat surprisingly when NaCl concentration was reduced in the pâté compared to the standard pâté, which contained 3.66% (w/v) NaCl in the water phase, *sigB* transcript levels were significantly increased in some strains (Olesen et al., 2010). Rantsiou et al. (2012b), suggest that temperature is the main variable contributing to the differences in *sigB* transcription that they observe. Differences in upregulation of expression of stress related genes and virulence genes have also been observed in other studies but often no particular pattern can be established (Duodu et al., 2010; Hadjilouka et al., 2016). Overall these studies show that different stresses encountered within foods can influence the induction of stress related genes and therefore have the potential to influence the gastrointestinal stages of a food-borne infection by *L. monocytogenes*.

### Overlap between Stress Response and Virulence

Tolerance to environmental stress and virulence can be considered to be overlapping facets of the biology of *L. monocytogenes* (O’Byrne and Karatzas, 2008). Firstly, without a robust stress response this pathogen would not be able to survive and persist in the food chain sufficiently well to allow it to gain access to a mammalian host. Secondly, the stresses encountered within the host, especially in the upper gastrointestinal tract, represent a significant barrier that must be overcome in order for *L. monocytogenes* to establish an infection. Particular challenges are presented by the acidic pH of the stomach, the osmolality and presence of bile in the ileum. As discussed earlier, (see Osmotic Shock and Low pH) *L. monocytogenes* has specific mechanisms for coping to acid and osmotic stress, some of which are under the control of σ^B^. This pathogen is also remarkably tolerant to bile. It can colonize the murine gall bladder (Hardy et al., 2004), aided by its bile salt hydrolase (BSH; Sue et al., 2004; Begley et al., 2005), a bile exclusion system called BilE (Sleator et al., 2005) and two efflux pumps (MdrM and MdrT; Quillin et al., 2011). The *bsh* gene and the *bilE* operon are both under σ^B^ control (Fraser et al., 2003; Sue et al., 2004; Begley et al., 2005), while the efflux pumps are under the control of BrtA, a TetR-type transcriptional regulator (Quillin et al., 2011).

Having survived the stresses imposed by the GI tract the next step in establishing an infection is the invasion of epithelial cells in the intestinal villi (Cossart and Toledo-Arana, 2008). Invasion of epithelial cells is dependent on a surface protein called internalin (encoded by the *inlA* gene) whose expression is dependent on σ^B^ (Kim et al., 2004a, 2005). It is interesting that the regulator of the GSR has been co-opted to participate in regulating the expression of a dedicated virulence gene and perhaps suggests that escape from the harsh conditions in the lumen of the gastrointestinal tract can be partly viewed as a response to stress (O’Byrne and Karatzas, 2008). The transcriptional regulator PrfA, a member of the Crp/Fnr family of regulators, is the master regulator controlling expression of virulence genes required for the intracellular stages of the infection caused by *L. monocytogenes* (reviewed in Scortti et al., 2007). PrfA expression is activated at 37°C by a thermal sensing switch in the 5′UTR region of the *prfA* transcript (Johansson et al., 2002) and is also influenced by the CodY transcriptional regulator under conditions where branched chain amino acid levels are low (Lobel et al., 2015). The activity of PrfA is also modulated post-translationally by an association with a ligand whose identity has been elusive for many years. Recently, however, glutathione was identified as an allosteric modulator of PrfA activity (Reniere et al., 2015).

A number of lines of evidence indicate that there is regulatory cross talk between PrfA and SigB but the precise nature of this link has been difficult to define (O’Byrne and Karatzas, 2008). A number of transcriptomic studies have identified sets of genes whose regulation is influenced both by PrfA and by σ^B^ (Kazmierczak et al., 2003; Milohanic et al., 2003; Ollinger et al., 2009; Toledo-Arana et al., 2009; Chaturongakul et al., 2011). σ^B^ contributes directly to the regulation of a number of virulence genes including the *inlAB* operon (Kim et al., 2004a, 2005) and *prfA* itself (Nadon et al., 2002; Rauch et al., 2005; Schwab et al., 2005). Although *prfA* is preceded by a σ^B^-dependent promoter (designated *prfAP2*) the significance of this promoter *in vivo* remains unclear since it overlaps with a σ^A^ promoter and it can be deleted without an obvious effect on haemolysis (Nadon et al., 2002). Overall it appears that the dominant role for σ^B^ is during the gastrointestinal stage of the infection (Garner et al., 2006) whereas PrfA dominates after the intestinal barrier has been breached (Toledo-Arana et al., 2009). But the multiple and complex regulatory inputs that exist to control PrfA expression and activity probably allow σ^B^-mediated fine tuning of the PrfA regulon under certain conditions.

## Methods of Controlling *L. monocytogenes*

Measures to control *L. monocytogenes* in the food chain mainly focus on the food processing environment, including personnel, and the formulation and processing of the product itself. Here we review some of the sanitizers that are in common use to control *L. monocytogenes* in food processing environments and consider some novel control strategies that are beginning to show promise and that might find application at different points in the food chain in the future.

### Sanitizers

Different sanitizers such as quaternary ammonium compounds (QACs), hydrogen peroxide, peracetic acid and sodium hypochlorite are often used for cleaning within food processing environments. It is known that these sanitizers are effective at killing planktonic *L. monocytogenes* cells (Kastbjerg and Gram, 2012; Ruckerl et al., 2014) and their effectiveness does not seem to differ between persistent and non-persistent strains of *L. monocytogenes* isolated from food environments (Magalhaes et al., 2016). Development of resistance against different sanitizers has also been investigated but the overall conclusion is that resistance does not seem to occur (Kastbjerg and Gram, 2012). Therefore no correlation between persistence and resistance to sanitizers has been discovered (Ruckerl et al., 2014). Different sanitizers have different mechanisms of inhibition. For example QACs such as benzalkonium chloride attack the cell membrane of cells, leading to cell leakage, while peracetic acid and sodium hypochlorite tend to act as oxidizing agents, creating reactive oxygen species (ROS) which lead to damage of cellular components (McDonnell and Russell, 1999).

To date, very little is known about the role of σ^B^ in the mechanism of resistance of *L. monocytogenes* to sanitizers. However, it has been observed that σ^B^ does play a role in the resistance of both planktonic and biofilm cells to benzalkonium chloride and peracetic acid over short periods of time (Ryan et al., 2009; van der Veen and Abee, 2010). Deletion of *sigB* reduces the levels of resistance against these sanitizers but does not affect growth in sub-lethal concentrations, while complementation of the mutation restores or even increases the resistance compared to the wild-type (van der Veen and Abee, 2010). While no studies have shown a correlation between σ^B^ and resistance to sodium hypochlorite, it is considered that it may have a role to play as genes involved in oxidative stress are under σ^B^ control (Ferreira et al., 2001; Boura et al., 2016). It is important to note that other systems controlled independently of σ^B^ (e.g., the efflux pumps QacH, MdrL, and Lde) have also been observed to impact survival in the presence of sanitizers such as benzalkonium chloride (Romanova et al., 2006; Muller et al., 2013, 2014).

### Photodynamic Inactivation

Alongside the discovery that several bacterial strains respond to light as a stress agent, interest has developed in the possible use of light as a bacterial containment method. Specifically, photodynamic inactivation (PDI) has been shown to be effective in the treatment of different bacteria, including antimicrobial resistant strains of bacteria (Maclean et al., 2010; Luksiene and Paskeviciute, 2011; Endarko et al., 2012; Murdoch et al., 2012; Bumah et al., 2015; Hosein et al., 2016). In the case of *L. monocytogenes*, light can decrease cell numbers in liquid culture, on surfaces and decrease its biofilm production meaning that PDI could be a very useful way of treating *L. monocytogenes* contamination in the food production environment (Murdoch et al., 2012; McKenzie et al., 2013; O’Donoghue et al., 2016). This treatment involves the use of a photosensitizer in combination with light and oxygen. The photosensitizer can be added to the medium or can be found naturally within cells in the form of endogenous molecules like porphyrins (Hamblin et al., 2005; Buchovec et al., 2010; Luksiene et al., 2010). The mechanism of PDI involves a photosensitizer becoming activated by the absorption of photons and this leads to the creation of a singlet state of the photosensitizer which can decay and omit fluorescence as it returns to the ground state, or it can form an excited triplet state. From this triplet state, photooxidation can occur via two different pathways leading to the formation of ROS or singlet oxygen (**Figure 2**; Sibata et al., 2001; Luksiene, 2003; Luksiene and Zukauskas, 2009; Robertson et al., 2009). The generation of ROS in response to light can lead to interactions with lipids and proteins within the cell membrane and also lead to DNA damage which can result in cell death (**Figure 2**). Addition of reactive oxygen scavengers to quench the effects of ROS has been shown to increase growth and survival of *L. monocytogenes* in the presence of blue light suggesting that ROS contribute to inhibition by visible light (Endarko et al., 2012; O’Donoghue et al., 2016). Interestingly, Tiensuu et al. (2013) found that many genes activated by Lmo0799 and σ^B^ in response to blue light, are involved in combating oxidative stress.

Gram-positive bacteria have been shown to be more susceptible to PDI than Gram-negative possibly due to differences in cell wall composition or due to different amounts of endogenous porphyrins being produced within the cell (Nitzan et al., 2004; Maclean et al., 2009). Many different bacteria including foodborne pathogens such as *L. monocytogenes, Bacillus cereus* and *Salmonella enterica* have been inhibited in various studies using visible light (Luksiene and Paskeviciute, 2011; Endarko et al., 2012; Murdoch et al., 2012; O’Donoghue et al., 2016). Endogenous porphyrins are produced through the heme biosynthetic pathway in bacteria and act as natural photosensitzers within the cell. Some studies have proposed boosting porphyrin production within cells by adding increased amounts of 5-aminolevulinic acid, a precursor of the heme biosynthetic pathway (Nitzan et al., 2004; Buchovec et al., 2010). Other studies have sought to test whether the addition of exogenous photosensitizers to the medium could increase the sensitivity of *Listeria* to PDI (Romanova et al., 2003; Luksiene et al., 2010; Lin et al., 2012). Luksiene and Paskeviciute (2011) successfully used light in combination with Na-chlorophyllin to reduce levels of contamination by *L. monocytogenes* on strawberries, proving that PDI could also be used in combination with approved food additives to control the growth and survival of *L. monocytogenes* on food products.

### Innovative Strategies for Reducing the Risk of *L. monocytogenes*

Strategies aimed at reducing the risk of listeriosis usually focus on the elimination of the organism at the stage of food processing as well as designing food preservation regimes that don’t support the growth of *L. monocytogenes*. Food preservation systems generally employ generic stress “hurdles” that act synergistically to inhibit microbial growth (Leistner, 2000), (e.g., reduced water activity combined with acidic pH), but the next generation of food preservatives might usefully target specific protective mechanisms and thereby prevent food pathogens from protecting themselves. As discussed in section “Nisin” is increasingly being used to prevent the growth of *L. monocytogenes* in food (reviewed in Cleveland et al., 2001). Its inhibitory mode of action is twofold; it interferes with cell wall biosynthesis and also disrupts the cytoplasmic membrane (McAuliffe et al., 2001). This dual action renders the cell vulnerable particularly when additional preservation-related stresses are also present in the food matrix. Lytic bacteriophages that target *L. monocytogenes* have also been considered for biocontrol of this pathogen. For example, broad host-range phages such as A511 and P100 have been shown to be effective at reducing viable *L. monocytogenes* cells to undetectable levels in some RTE foods (Carlton et al., 2005; Guenther et al., 2009; Bigot et al., 2011). In the future it might also be possible to target the regulators that control stress tolerance (σ^B^) and virulence (PrfA). A small molecule that blocks σ^B^ activity and reduces host cell invasion has recently been described (Palmer et al., 2011). The compound, 2 fluoro-phenyl-styrene-sulfonamide (FPSS), apparently blocks the release of σ^B^ from its anti-sigma factor RsbW, thereby preventing it from participating in transcription (Ringus et al., 2013), but the precise mode of action has not yet been established. Blocking the expression of virulence functions might also be a viable means of reducing the risk to food consumers. Recently a class of ring-fused 2-pyridone molecules have been identified that bind to PrfA and decrease its affinity for its consensus binding site on DNA (Good et al., 2016). A structural analysis of the interaction of PrfA with one of these molecules revealed that it interacts at two different sites on the protein that could prevent both allosteric activation of PrfA and also the correct alignment of the DNA binding helix-turn-helix domain, thereby interfering with its ability to stimulate virulence gene expression. Additional work will be needed to develop these molecules further as potential therapeutic agents or even as designer food-preservatives.

## Concluding Remarks

While *L. monocytogenes* continues to present a very real risk to human health we now have a greatly improved understanding of its ecology, genetics and physiology. The ability to rapidly identify the sources of contamination using the latest genetic typing methods (including whole genome sequencing) means that food producers will better know where to target their efforts at reducing the occurrence in foods. Our understanding of the biology of *L. monocytogenes*, including a detailed knowledge of the protective strategies it uses to defend itself against harsh conditions, should better equip us to design food processing and preservation strategies that target the organisms Achilles’ heel. There are still significant gaps in our knowledge, not least of which concerns the precise mechanisms that *L. monocytogenes* uses to sense its environment and how it couples its stress response to its pathogenicity, but the impressive research activity in these fields is likely to produce answers to these questions in the near future. The prevalence of this pathogen in the natural environment means that eliminating it from the food chain is almost impossible so reducing its occurrence in food through sound processing practices and carefully designed preservation strategies is the best approach for reducing the risk to food consumers. Novel approaches like inactivation with visible light or using inhibitors designed to target the regulatory machinery of this pathogen show great promise and are likely to be adopted in the years ahead.

## Author Contributions

All authors listed, have made substantial, direct and intellectual contribution to the work, and approved it for publication.

## Conflict of Interest Statement

The authors declare that the research was conducted in the absence of any commercial or financial relationships that could be construed as a potential conflict of interest.
